# Diet Change Improves Obesity and Lipid Deposition in High-Fat Diet-Induced Mice

**DOI:** 10.3390/nu15234978

**Published:** 2023-11-30

**Authors:** Tengteng Ji, Bing Fang, Fang Wu, Yaqiong Liu, Le Cheng, Yixuan Li, Ran Wang, Longjiao Zhu

**Affiliations:** Key Laboratory of Precision Nutrition and Food Quality, Department of Nutrition and Health, China Agricultural University, Beijing 100193, China

**Keywords:** obesity, diet change, weight loss, fat deposition, adipose stem cells

## Abstract

The number of obese people is increasing dramatically worldwide, and one of the major causes of obesity is excess energy due to high-fat diets. Several studies have shown that reducing food and energy intake represents a key intervention or treatment to combat overweight/obesity. Here, we conducted a 12-week energy-restricted dietary intervention for high-fat diet-induced obese mice (C57BL/6J) to investigate the effectiveness of diet change in improving obesity. The results revealed that the diet change from HFD to NFD significantly reduced weight gain and subcutaneous adipose tissue weight in high-fat diet-induced obese mice, providing scientific evidence for the effectiveness of diet change in improving body weight and fat deposition in obese individuals. Regarding the potential explanations for these observations, weight reduction may be attributed to the excessive enlargement of adipocytes in the white adipose tissue of obese mice that were inhibited. Diet change significantly promoted lipolysis in the adipose tissue (eWAT: *Adrb3*, *Plin1*, *HSL*, and *CPTA1a*; ingWAT: *CPT1a*) and liver (reduced content of nonesterified fatty acids), and reduced lipogenesis in ingWAT (Dgat2). Moreover, the proportion of proliferative stem cells in vWAT and sWAT changed dramatically with diet change. Overall, our study reveals the phenotypic, structural, and metabolic diversity of multiple tissues (vWAT and sWAT) in response to diet change and identifies a role for adipocyte stem cells in the tissue specificity of diet change.

## 1. Introduction

Overweight/obesity caused by energy-intensive foods and sedentary lifestyles is rapidly increasing in prevalence worldwide [1]. Indeed, it is projected that 51% of the global population will be overweight or obese by 2035. A chronic high-fat diet leads to excess energy in the organism, which is stored in the form of triglyceride (TG) in white adipose tissue [2]. In the long run, when the organism develops obesity, the white adipose tissue becomes dysfunctional and is unable to store too much TG. This leads to lipid overflow and triggers ectopic fat deposition in other tissues, including the liver and muscle, which causes metabolic disorders. The organism produces dyslipidemias, including increased TG, and non-esterified fatty acid (NEFA) [3,4]. Metabolic disturbances are closely related to cardiovascular disease, type 2 diabetes, dementia, and cancer [5,6]. Therefore, obesity negatively affects most physiological functions and almost all organ systems, thereby increasing mortality and reducing human life expectancy by 5–7 years [7].

The cause of obesity is excess energy caused by a high-fat diet, and several studies have shown that reducing food intake and energy intake can be used as a key intervention or treatment against overweight/obesity. A previous study in obese patients revealed that a low-fat diet resulted in weight loss of 8 kg in 6 months [8] and significantly restored obesity-related metabolic dysfunctions, including reduced visceral fat mass [9], improved glucose homeostasis, and hepatic ectopic fat deposition [10,11,12]. Diet change-mediated prevention is also widely available in other aspects, which can significantly reduce the occurrence and/or progression of type 2 diabetes, nephropathy, cardiomyopathy, neurodegenerative diseases, and several autoimmune diseases [13,14,15,16,17]. Such preventative measures can also improve the learning and memory deficits induced by a high-fat diet [18,19] and extend lifespan by preventing chronic diseases and maintaining metabolic and biological functions in a more youthful state [11,20,21]. However, numerous population and animal studies have shown that long-term energy restriction is challenging, revealing a weight loss of only 3–4 kg in 3 years [22], much less than that in a shorter time of 6 months [8]. Although short-term energy restriction is effective for weight loss, it results in faster weight recovery following re-exposure to a high-fat diet and even causes weight regain and fat mass gain [23,24,25,26,27]. Several studies have reported that regaining weight and recurrence of metabolic complications within 12 months of initial weight loss may be even worse; this may be related to a progressive increase in the high-/low-fat diet cycles, which accelerates the susceptibility to accelerated weight regain and its associated metabolic complications [26,28]. The mechanism behind both weight regain and the recurrence of metabolic complications may be closely related to changes in the number and function of adipocytes during obesity caused by adipose tissue overexpansion, which in turn leads to changes in the energy response [29].

Here, to investigate the effects of energy restriction as a nutritional intervention of the high-fat diet, we conducted a 12-week energy restriction on 21-week high-fat diet-induced obese mice and investigated the effect of reversing diet on the fat deposition and glucolipid metabolic response function of stem cells in obese mice. This study provides theoretical support for the effectiveness of diet change in weight loss and metabolic function in obese people and provides new insights into the mechanism of these bounce-back effects.

## 2. Materials and Methods

### 2.1. Animals and Experimental Design

All of the experimental protocols were approved by the Institutional Animal Care and Ethics Committee of China Agricultural University (AW21403202-5-1). Three-week-old male C57BL/6J mice (*n* = 18) were purchased from SPF (Beijing, China) Biotechnology Co. Ltd. and domesticated for 1 week. All of the mice were housed in static cages at room temperature (26 °C) under a 12 h light-dark cycle. Mice were single caged. The animals were randomly grouped at the beginning and there was no significant difference in the initial body weights of the animals in each group. Body weight and food intake were monitored weekly throughout the study while allowing ad libitum access to food and water. Mice were randomly divided into two feeding groups: (1) normal-fat diet (NFD, *n* = 6, 11% energy from fat; AIN-93G, Research Diets, Beijing, China) and (2) high-fat diet (HFD, *n* = 12, 60% energy from fat; D12492, Research Diets, Beijing, China). The composition of the diet is listed in Table 1. After 21 weeks on their respective diets, mice from the HFD group were randomly divided into two groups: six mice were kept on the HFD, and the remaining mice were switched to the normal-fat diet (H-NFD, *n* = 6). All of the mice were euthanized by carbon dioxide inhalation and cervical dislocation after 12 weeks of continued feeding. Blood samples, liver, white adipose, tissue including epididymal (eWAT), perirenal (pWAT), inguinal (ingWAT), axilla (aWAT), and brown adipose tissue (BAT), were rapidly collected from individual mice.

### 2.2. Intraperitoneal Glucose Tolerance Test

Mice were injected intraperitoneally with 1 g/kg glucose solution after 14 h of fasting. Blood glucose was measured from a drop of tail blood at 0, 15, 30, 60, 90, 120, and 180 min using a glucometer (Roche, ACCU-CHEK Performa, Germany). Then, the area under the glucose tolerance test (GTT) curve (AUC) was evaluated by measuring the AUC and subtracting it from the baseline [30].

### 2.3. Assessment of Energy Metabolism

The OxyletPro system was used to measure respiratory metabolism, including O_2_ consumption (VO_2_) and CO_2_ production (VCO_2_), and calculate the respiratory quotient (RQ) and energy expenditure (EE). Mice were individually housed and maintained in a metabolic chamber for 24 h to acclimatize to the experiments. Formal experiments followed lasting 48 h. Data were collected continuously for 48 h, during which time, a 12-h light/dark cycle was maintained [31].

### 2.4. Isolation of the Stromal Vascular Fraction (SVF)

Subcutaneous (sWAT, including ingWAT and aWAT) and visceral adipose tissue (vWAT, including eWAT and pWAT) depots from each mouse were dissected in ice-cold phosphate buffered saline (PBS). The tissue was cut with scissors, transferred to collagenase II (Sigma-Aldrich, St. Louis, MO, USA, #C6885-1G, sWAT, 2 mg/mL; vWAT, 1 mg/mL collagenase buffer [PBS]), digested for 30 min at 37 °C, with shaking at 220 rpm and vortexing for 15 s every 10 min. The digestion was terminated with high-glucose DMEM (Vivacell, Yerevan, Armenia, #06-1055-1ACS) containing 10% fetal bovine serum (Vivacell, #C04001-500). The cell suspension was filtered through a 70-μm cell strainer and centrifuged at 500× *g* for 5 min. Floating mature lipid-filled adipocytes were aspirated, and red blood cell lysis buffer (Solarbio, Beijing, China, #R1010) was added to the cell sediment to break down the cell clumps. After 5 min, DMEM was added to complete the incubation. Subsequently, the cell suspension was filtered through a 40 μm cell strainer and centrifuged at 500× *g* for 5 min. Finally, the supernatant was discarded and 1 mL of PBS was added to resuspend the cells [32].

### 2.5. Flow Cytometry Sorting

The above cell suspensions were supplemented with the following fluorophore-conjugated antibodies: Alexa Fluor^®^ 488 anti-mouse CD31, Alexa Fluor^®^ 488 anti-mouse CD45, Alexa Fluor^®^ 488 anti-mouse TER-119 (Biolegend, San Diego, CA, USA, #303110, #304017 and #116215, respectively) to select the Lin^−^ population; PE/Cyanine7 anti-mouse Sca-1 (Biolegend, #122514) and PE/Dazzle™ 594 anti-mouse CD34 (Biolegend, #119330) to enrich the Lin^−^ population with adipose stem cells (ASCs); APC mouse anti-mouse CD36 (BD, #562744), PE anti-mouse CD26 (DPP4) (Biolegend, #137804), and PerCP/Cyanine5.5 anti-mouse CD54 (Biolegend, #116124) to separate proliferative or differentiated ASCs. Cells were incubated with the antibody cocktail for 30 min on ice, protected from light, and subjected to fluorescence-activated cell sorting (FACS) using a Becton Dickinson, Franklin Lakes, NJ, USA, FACSAria TM III [32].

### 2.6. Histological Analysis

Tissues were fixed in a 4% paraformaldehyde medium and embedded in paraffin. Two consecutive sections (3-μm) were cut from each tissue and stained with hematoxylin and eosin (Solarbio, #G1100 and #G1080). Three mice from each group were randomly selected; two sections from each mouse and three images from each section were randomly collected for analysis. A Leica DM6B upright microscope equipped with a 10× or 20× objective was used for image acquisition. Quantification of adipocyte diameters was performed using Image-Pro Plus 6.0 software (Media Cybernetics, Rockville, MD, USA).

### 2.7. Oil Red O Staining

Mouse livers were fixed in 4% paraformaldehyde, embedded in paraffin, cut into sections (10 μm), and stained with hematoxylin and eosin (Solarbio, #G1100 and #G1080). Frozen sections of paraformaldehyde-fixed liver were stained with Oil red O solution (Solarbio, #G1260).

### 2.8. Protein Expression Analysis

Specimens were homogenized in ice ice-cold buffer containing RIPA (Beyotime Biotechnology, Shanghai, China, #P0013B) and protease inhibitors (Solarbio, #A8260). The homogenates were centrifuged at 16,000× *g* for 10 min at 4 °C, and the supernatants were set aside for subsequent experiments. Protein concentrations were determined by the bicinchoninic acid method (Pierce). Protein lysates were subjected to SDS-PAGE and then transferred to PVDF membranes (Millipore, #ISEQ00010). Subsequently, the membranes were incubated overnight at 4 °C, along with specific primary antibodies against anti-perilipin-1 (*Plin1*, Cell Signaling Technology, #9349, 1:2000), hormone sensitive lipase (*HSL*, Cell Signaling Technology, #4107, 1:4000), phosphor-*HSL* (Ser660) (Cell Signaling Technology, #45804, 1:2000), Akt (Cell Signaling Technology, #9272, 1:4000), phosphor-Akt (Ser473) (Cell Signaling Technology, #4060, 1:2000), and PPARg (Cell Signaling Technology, #2443, 1:1000). For secondary antibody incubation, anti-rabbit HRP (Promega, Madison, WI, USA) was diluted in TBS-T containing 5% milk, followed by enhanced chemiluminescence detection and quantification of the band density. Blotted membranes were probed with monoclonal antibodies against β-tubulin (Immunoway, #YM3030, 1:10,000) to assess protein loading and to normalize target protein levels to β-tubulin levels.

### 2.9. Gene Expression Analysis

Total RNA was extracted mainly from eWAT and ingWAT using TRIzol reagent (Invitrogen, Waltham, MA, USA, #15596026). Total RNA (1500 ng) was then reverse transcribed into cDNA using All-In-One 5X RT Mastermix (Abm, #G492). Gene expression was analyzed by quantitative real-time PCR (qPCR) using SYBR Green (Takara, Kusatsu, Japan, #RR82LR) and normalized to GAPDH. The primer pair sequences are shown in Table 2.

### 2.10. Biochemical Analysis

The levels of triglyceride and NEFA in the eWAT, ingWAT, and liver were determined using a colorimetric kit (Nanjing Jiancheng Bioengineering Institute, Nanjing, China, #A110-1-1; Boxbio, #AKFA008M). Serum insulin levels were determined using an Ultra-Sensitive Mouse Insulin ELISA Kit (Crystal Chem, Elk Grove Village, IL, USA 90080, Beijing, China).

### 2.11. Statistical Analysis

Statistical comparisons were made by one-way analysis of variance followed by post hoc Tukey’s multiple comparison test using GraphPad Prism version 9.5.0 software. Significance was defined as a *p*-value < 0.05 (indicated by * or ^#^) or 0.01 (indicated by ** or ^##^). Data are presented as the mean ± SEM of the group.

## 3. Results

### 3.1. Change to a NFD Immediately Alleviates HFD-Induced Body Weight and Fat Gain

**Figure 1:**
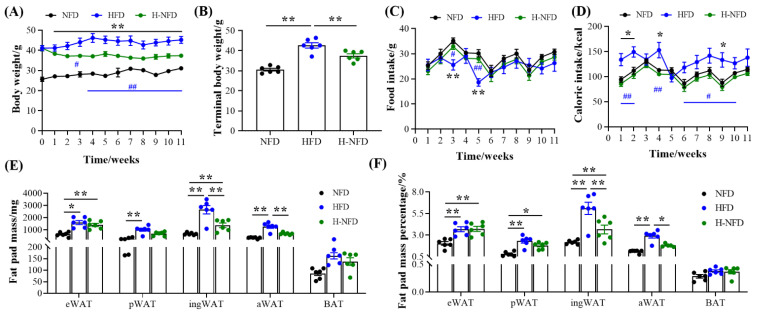
Change to a normal-fat diet (NFD) immediately alleviates high-fat diet (HFD)-induced body weight and fat gain. (**A**) Body weight, (**B**) terminal body weight, (**C**) food intake, (**D**) caloric intake, (**E**) fat mass, and (**F**) the percentage of eWAT, pWAT, ingWAT, aWAT, and BAT of mice in NFD, HFD, and H-NFD groups (*n* = 6). Values are expressed as the mean ± s.e.m. of biologically independent samples. *p*-values were determined using unpaired *t*-tests. *^/#^ *p* < 0.05, **^/##^ *p* < 0.01 (*NFD vs. HFD; #HFD vs. H-NFD).

Throughout the experiment, when converting a HFD to NFD, the body weight decreased quickly, showing a significant difference from the third week until the end of the experiment (Figure 1A). At the end of the experiment, the body weight of the H-NFD group decreased significantly by 12.22% compared to that of the HFD group (Figure 1B) but did not decrease to the level observed in the NFD group mice. Meanwhile, there was no significant change in food intake among the three groups, except for in the third and fifth weeks (Figure 1C), when the mice in the HFD group showed a significant decrease in food intake. The large drop in food intake observed in the fifth week may be due to the stress associated with changing cages in the animal house, after which the mice gradually acclimatized and recovered, both in terms of food intake and caloric intake (Figure 1C,D), but their body weight did not change significantly. On this basis, we counted the energy intake of the three groups of mice, the results of which revealed that the mice in the NFD and H-NFD groups showed a 25% reduction in caloric intake compared to those in the HFD group (Figure 1D), which may explain why the mice in the HFD group were fatter than those in the NFD group; this may also be the underlying reason for the significant reduction in body weight of the obese mice after the change from the HFD to NFD (i.e., the H-NFD group). In addition, when assessing adipose tissue deposition, the weight and percentage of sWAT (including ingWAT and aWAT) were significantly decreased in the H-NFD group compared to those in the HFD group, whereas the vWAT was unchanged (Figure 1E,F). Among the sWAT, the weight and percentage of aWAT regressed to the level of the NFD group mice (Figure 1E,F).

**Figure 2:**
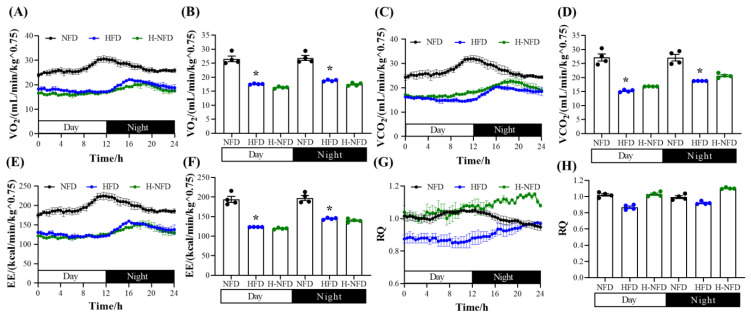
Change to NFD did not reverse HFD-induced low energy expenditure. (**A**) Oxygen consumption (VO_2_) over 24 h, and (**B**) the quantitative mean values during the day- and night-time. (**C**) Carbon dioxide production (VCO_2_) over 24 h, and (**D**) the quantitative mean values during the day- and night-time. (**E**) Whole-body energy expenditure (EE) over 24 h, and (**F**) the quantitative mean values during the day- and night-time. (**G**) Respiratory quotient over 24 h, and (**H**) the quantitative mean values during the day- and night-time, which is defined as the ratio of carbon dioxide production and oxygen consumption simultaneously (*n* = 4). Values are expressed as the mean ± s.e.m. of biologically independent samples. *p*-values were determined using unpaired *t*-tests. * *p* < 0.05.

We also conducted experiments related to energy metabolism. The results revealed that the HFD-induced obese mice had significantly lower VO_2_, VCO_2_, and energy expenditure in both the light and night phases. Excessive energy intake and low energy expenditure led to HFD-induced obesity in mice. In contrast, the change from a HFD to NFD did not result in any improvement in low O_2_ consumption, low CO_2_ production, or low energy expenditure (Figure 2A–F). Additionally, the RQ did not change significantly between the three groups (Figure 2G,H).

### 3.2. Change to NFD Decreases Adipocyte Size

**Figure 3:**
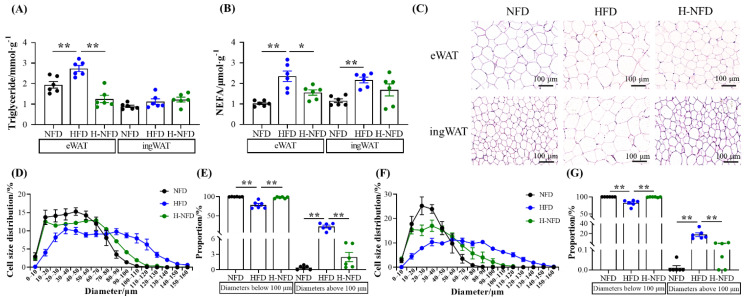
Change to NFD alleviates HFD-induced hypertrophy in both the visceral and subcutaneous adipose tissue. (**A**) Triglyceride and (**B**) non-esterified fatty acid (NEFA) contents in eWAT and ingWAT. (**C**) Representative images of hematoxylin and eosin (H&E) staining of eWAT and ingWAT (20 × magnification; scale bars: 0.1 mm). (**D**) The distribution of adipocytes diameters in eWAT. The adipocyte size was measured using Image-Pro Plus software; six fields were chosen for each mouse and 50–80 cells were counted for each field. (**E**) Proportion of adipocytes with diameters >100 μm in eWAT. (**F**) The distribution of adipocyte diameters in ingWAT. Adipocyte size was measured using Image-Pro Plus software; six fields were chosen for each mouse and 50–80 cells were counted for each field. (**G**) Proportion of adipocytes with diameters > 100 μm in ingWAT. (*n* = 6). Values are expressed as the mean ± s.e.m. of biologically independent samples. *p*-values were determined using unpaired *t*-tests. * *p* < 0.05, ** *p* < 0.01.

HFD-induced obese mice exhibited high concentrations of triglycerides and NEFA in both eWAT and ingWAT. The change from a HFD to NFD resulted in a significant decrease in triglyceride and NEFA content in eWAT, which was accompanied by no significant changes in ingWAT (Figure 3A,B). Additionally, hematoxylin and eosin (HE)-stained sections showed that adipocytes in NFD mice were small in diameter, while those in HFD mice expanded significantly. The change from a HFD to NFD reversed the expansion of adipocytes (Figure 3C), with the proportion of adipocytes with diameters >100 μm decreasing from 21.72% and 17.88% to 2.46% and 0.58% in eWAT and ingWAT, respectively, and the proportion of adipocytes with diameters <100 μm increasing from 78.28% and 82.18% to 97.54% and 99.42%, respectively (Figure 3D–G).

### 3.3. Change to NFD Enhances Lipolysis in Adipose Tissue

**Figure 4:**
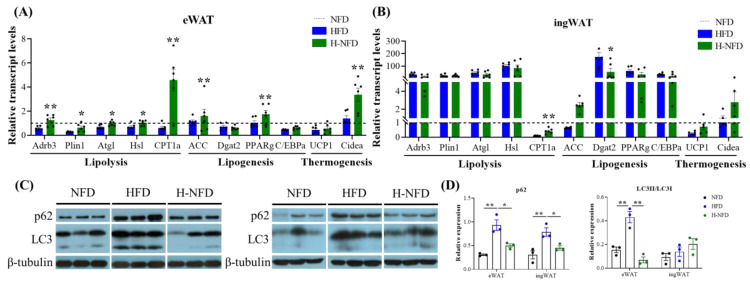
Change to NFD activates enzyme involved in lipolysis and clearance of hypertrophic adipocytes. (**A**,**B**) Relative mRNA expression of genes involved in lipolysis (e.g., *Adrb3*, *Plin1*, *Atgl, HSL*, *CPT1a*), lipogenesis (e.g., *ACC*, *Dgat2*, *PPARg*, *C/EBPa*), and thermogenesis (*UCP1*, *Cidea*) in (**A**) eWAT and (**B**) ingWAT measured by quantitative real-time PCR (*n* = 6). (**C**) Immunoblots of p62 and LC3 in eWAT (left) and ingWAT (right). (**D**) The quantitative western blot results in eWAT (left) and ingWAT (right). Data are expressed as the mean ± s.e.m. of biologically independent samples (*n* = 3). *p*-values were determined using unpaired *t*-tests. * *p* < 0.05, ** p < 0.01.

HFD-induced obese mice have reduced expression of lipolysis-related genes in eWAT and increased expression levels of lipolysis- and lipogenesis-related genes in ingWAT. Change of a HFD to NFD significantly increased the mRNA expression of beta-3-adrenergic receptor (*Adrb3*), perilipin 1 (*Plin1*), adipose triglyceride lipase (Atgl), hormone-sensitive lipase (*HSL*), and carnitine palmitoyltransferase 1a (*CPT1a*) in eWAT and *CPT1a* in ingWAT, all of which are associated with lipolysis (Figure 4A,B). However, the mRNA expression of acetyl-coenzyme A carboxylase alpha (ACC) and peroxisome proliferator-activated receptor γ (PPARg) was significantly increased in eWAT, whereas the mRNA expression of diacylglycerol-O-acyltransferase 2 (Dgat2) and CCAAT enhancer binding protein alpha (C/EBPa) was unchanged (Figure 4A,B). The ingWAT that showed a reduction in weight also exhibited a dramatic decrease in the expression of Dgat2, which was accompanied by no significant change in mRNA expression of the adipogenesis-related genes ACC, PPARg, and C/EBPa (Figure 4A,B). In addition, the expression of cell death-inducing DFFA-like effector a (Cidea), a thermogenesis-related gene, was significantly increased in eWAT (Figure 4A). We also examined autophagy-related protein expression using western blot (WB), the results of which revealed a significant elevation in p62 and LC3 protein expression in the eWAT of HFD-induced obese mice, whereas the expression was reduced after the change from a HFD to NFD. Meanwhile, the expression of p62 protein in ingWAT was consistent with the changes in eWAT among the three groups. (Figure 4C,D).

### 3.4. Change to a NFD Reduces Ectopic Fat Deposition in the Liver and Worsened Glucose Homeostasis

**Figure 5:**
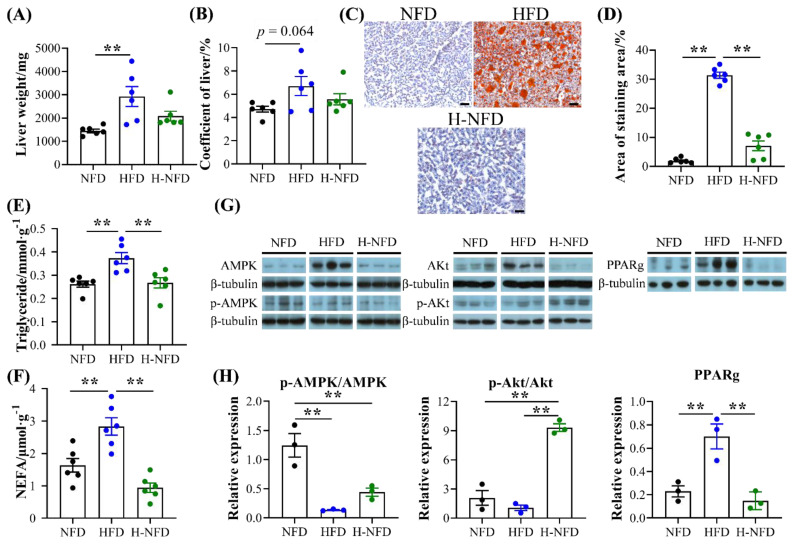
Change to NFD alleviates HFD-induced lipid accumulation in the liver. (**A**) Liver weight. (**B**) Liver coefficient. (**C**) Representative images of hematoxylin and eosin (H&E) staining of liver (10 × magnification; scale bars: 0.1 mm). (**D**) Area ratio of Oil Red O staining area measured using Image-Pro Plus software (*n* = 6). (**E**) Triglyceride and (**F**) non-esterified fatty acid (NEFA) contents in the liver (*n* = 6). (**G**) Immunoblots of AMPK, p-AMPK, Akt, p-Akt, and PPARg in the liver, and (**H**) quantitative results in the liver (*n* = 3). Data are expressed as the mean ± s.e.m. of biologically independent samples. *p*-values were determined using unpaired *t*-tests. ** *p* < 0.01.

The liver weight of HFD-induced obese mice was significantly higher than that of mice on a NFD, although there was no difference in the liver coefficient (Figure 5A,B). Additionally, obesity-induced ectopic deposition of fat in the liver by oil red O staining, whereas fat deposition was reduced by 24.26% after the change from a HFD to NFD (Figure 5C,D), in addition to a significant reduction in triglyceride and NEFA content in the liver (Figure 5E,F). The results of WB showed a significant increase in the relative expression of p-Akt/Akt in the liver after the change from a HFD to NFD, which was accompanied by a significant decrease in PPARg (Figure 5G,H).

**Figure 6:**
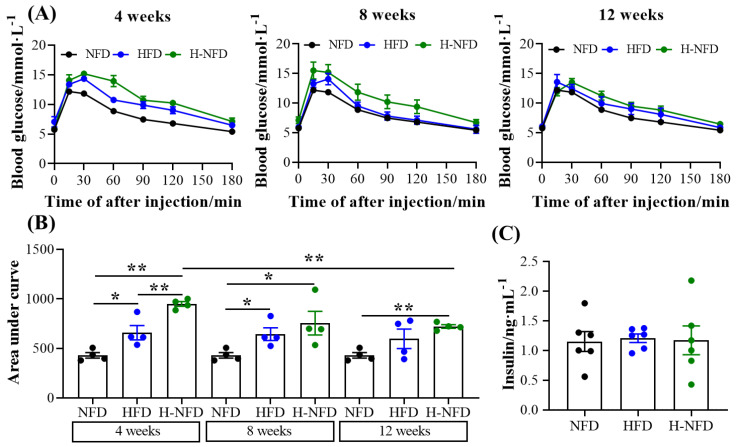
Change to NFD did not improve HFD-induced glucose intolerance. (**A**) IPGTT graphs after 4, 8, and 12 weeks. (**B**) Area under the baseline curve in the IPGTT graph (*n* = 4). (**C**) Serum contents of insulin at the end of the experiment (*n* = 6). Values are expressed as the mean ± s.e.m. of biologically independent samples. *p*-values were determined using unpaired *t*-tests. * *p* < 0.05, ** *p* < 0.01.

In terms of glucose metabolism, HFD-induced obese mice exhibited significant glucose intolerance (Figure 6A,B). Instead of beneficially affecting glucose tolerance, the change from a HFD to NFD worsened glycemic control, but the degree of deterioration gradually decreased over time (Figure 6A,B). Furthermore, at the end of the experiment, there was no significant difference in circulating insulin levels between the three groups (Figure 6C).

### 3.5. Change to a NFD Partially Reverse the Microenvironment of Adipose Tissue

**Figure 7:**
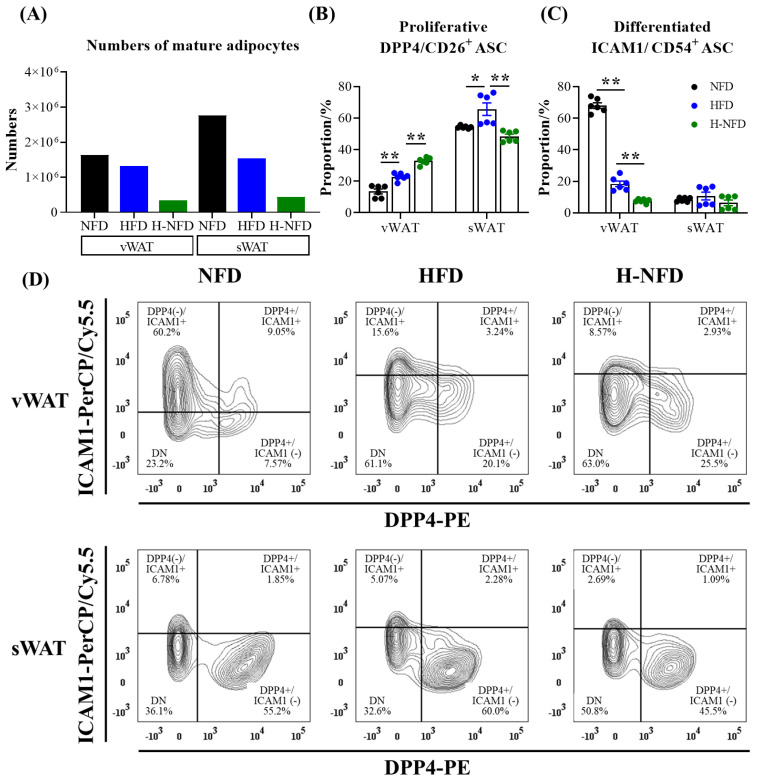
Change to NFD reduces HFD-induced proliferation and differentiation of ASCs. The number of mature adipocytes (**A**) (*n* = 1). (**B**,**C**) The proportion of different types of ASCs, including (**B**) proliferative DPP4/CD26^+^, (**C**) differentiated ICAM1/CD54^+^ ASCs (*n* = 6), and (**D**) their representative FACS plots. Data are expressed as the mean ± s.e.m. of biologically independent samples (*n* = 6). *p*-values were determined using unpaired *t*-tests. * *p* < 0.05, ** *p* < 0.01.

The numbers of primary adipocytes in the HFD group were less than those in the NFD group, which were further decreased following the change from a HFD to NFD, especially in sWATs (Figure 7A). According to the identified types of ASCs, a HFD significantly elevated the proportion of the proliferative (DPP4/CD26^+^) ASCs in both sWAT and vWAT. In contrast, change to a NFD further increased the proliferative ASCs in vWAT, but in sWAT, decreased the proportion to the level observed in the NFD group. A HFD significantly decreased the proportion of differentiated (ICAM1/CD54^+^) ASCs, and NFD change further reduced the proportion in vWAT; neither the HFD nor change to a NFD influenced these ASCs in sWAT (Figure 7C,D).

## 4. Discussion

With the change in modern lifestyle, excessive energy intake and sedentary lifestyle are common, which leads to excess energy in the organism and triggers obesity [33]. Several studies have shown that energy restriction improves obesity-related metabolic dysfunction [8,9,10,11,12], but some studies have also shown that recurrence of metabolic complications may occur during weight loss [26,28]. Therefore, the aim of this study was to investigate the effects of energy restriction as a nutritional intervention on a high-fat diet.

It has been reported that a low-fat diet helps to control body weight [18]. Our findings confirm this. Our results demonstrated that the diet change from HFD to NFD (approximately 25% energy restriction, Figure 1D) significantly reduced weight gain and adipose tissue weight in HFD-induced obese mice, which is consistent with the findings of Vatarescu and Hinder et al. [10,34].

Adipose tissue, as a dynamic organ distributed throughout the body, has a high degree of plasticity, which is reflected in its structural and metabolic aspects. First, adipose tissue has a remarkable ability to expand and contract in response to different physiological challenges. During obesity, adipocytes store excess energy by increasing in size and number, which is confirmed by our HE staining results (Figure 3C) [35,36]. The diet change from HFD to NFD can be regarded as “undernutrition” compared to a HFD, where adipocytes cope with energy changes by reducing excessive enlargement in HFD conditions (Figure 3C). Depending on the site of deposition, the white adipose tissue is mainly categorized into vWAT and sWAT [37]. After the diet change from HFD to NFD, although both types of adipocyte over-enlargement were significantly reduced, the triglyceride and NEFA contents were significantly changed only in eWAT (Figure 3A,B). This may be due to the higher catabolic and metabolic activity of vWAT compared to sWAT, which also corresponds to the lipolysis-related genes. sWAT, as an energy buffer depot, is mainly responsible for converting excess energy into fat and storing it, with a lower risk of metabolic abnormalities, and is more inclined to adipogenesis [38,39]. This is also the primary reason for the concentration of fat in sWAT in obese mice (Figure 1E).

In addition, adipose tissue plasticity during obesity is reflected in the response to organismal energy metabolism by increasing lipogenesis (energy storage) and decreasing lipolysis (energy release) [35]. The energy-substrate demand during food deprivation caused by the diet change from HFD to NFD serves as the primary driver of induced lipolysis [40], facilitating lipolysis and releasing fatty acids, which contributes to the use of fatty acids by tissues, including white adipose tissue and liver. This process accelerates weight loss in adipose tissue and reduces ectopic fat deposits in the liver (Figure 1E and Figure 5C). In addition, the qPCR results demonstrated that eWAT lipolysis increased after the change from a HFD to NFD, and the degree of response to lipolysis was greater than that to ingWAT (Figure 4A,B). *Plin1*, a protein on the surface of adipocyte lipid droplets that facilitates the entry of lipase into these droplets [41], was first activated, followed by the up-regulation of the expression of the rate-determining lipolytic enzymes ATGL, *HSL*, and *CPT1a*, which accelerated lipolysis in eWAT [42,43,44]. In contrast, the expression of eWAT lipogenesis-related genes was elevated (Figure 4A), including the rate-limiting enzyme for fatty acid synthesis (ACC) and the rate-determining enzyme for lipid accumulation-PPARg. The significant elevation of both resulted in no significant effect on the weight of vWAT (Figure 1E). Dgat2, the main enzyme catalyzing triglyceride synthesis, was significantly downregulated in ingWAT (Figure 4B), resulting in a significant decrease in sWAT weight (Figure 1E). Cidea is known to play an important role in regulating thermogenesis and lipolysis [45,46]. After the change from a HFD to NFD, although Cidea was significantly upregulated in eWAT (Figure 4A), it was insufficient to decrease the final fat mass. Taken together, these results suggest that the decreased gene expression described above can be explained by the results for fat mass. These findings also suggest that the diet change from HFD to NFD slows obesity by modulating a range of different enzymes and proteins to reduce white fat deposition, which in turn affects lipolysis and lipogenesis.

In addition to the above-mentioned lipolysis, regarding the catabolism of lipid droplets, lipophagy also plays a role in the removal of lipids [47,48,49,50]. In obesity, the expression of autophagy genes is upregulated and autophagy is activated, and this obesity-induced apoptosis of adipocytes promotes inflammation and insulin resistance [51,52]. It has been suggested that the upregulation of autophagy by obesity is likely to be a response to ER stress and is related to the degree of obesity and adipose distribution [53]. In addition, increased levels of LC3-II, a marker protein for autophagy, have been found in adipose tissues isolated from obese populations or obese mice, suggesting that autophagy is activated in obese populations and mice. This is consistent with our experimental results. The reason for activation is to promote adipogenesis to store more energy by activating autophagy in times of overnutrition [54]. Conversely inhibiting autophagy resists diet-induced obesity and reduces the accumulation of lipids in white adipocytes [55]. In our experiments, the expression of p62 and LC3 in adipose tissues is significantly down-regulated following the change from a HFD to NFD (Figure 4C,D), which counteracts the lack of controlled adipogenesis and prevents weight gain. This is consistent with the findings of Nunez et al. [56]. Thus, it seems that exploring the deep relationship between obesity and autophagy in adipocytes may provide potential targets for the prevention and treatment of obesity in the future.

Although diet change from HFD to NFD significantly improved weight gain, fat expansion, and balanced lipogenesis/lipolysis in obese mice, neither low energy metabolism nor abnormal glucose tolerance improved, while glycemic control worsened. It is possible that the effect of dietary therapy on glycemic control worsens with the relaxation of energy restriction, even in the presence of partial weight maintenance [28]. However, the underlying causes need to be explored. Based on this, we turned our attention to ASCs. In recent years, differences between adipose stem cells from vWAT and sWAT may be responsible for the observed functional differences between the two tissue types. ASCs produce adipocytes with different lipolytic capacities and lipogenic differentiation potentials [57]. A HFD significantly increased the proportion of proliferative (DPP4/CD26+) ASCs in both sWAT and vWAT, which is consistent with the results of Stefkovich et al. [58]. Upon change to a NFD, the proportion of proliferative ASCs was further increased in vWAT, but the proportion of proliferative ASCs in sWAT decreased to the level of the NFD group (Figure 7). This also corresponds to the results of fat mass observed in this experiment. We speculate that these results are due to the higher catabolic and metabolic activities and higher risk of metabolic abnormalities in vWAT, which serve to induce alterations in its stem cells to influence metabolic disturbances (glucose tolerance) in the body.

## 5. Conclusions

Our results revealed that dietary fat and energy restriction significantly reduced body weight, subcutaneous fat, and obesity-induced ectopic fat deposition in the livers of obese mice. More importantly, we identified an array of changes in ASCs in tissues following diet change (proliferative ASCs increased in vWAT and decreased in sWAT), suggesting different molecular mechanisms of energy response in visceral versus subcutaneous adipose tissue. However, neither the reduced energy metabolic rate nor abnormal glucose tolerance improved in obese mice, suggesting that reducing energy or fat intake alone is ineffective in preventing and controlling obesity-induced abnormal glucose tolerance. This means that considerably more work will need to be conducted to explore the improvement of metabolic disorders brought about by obesity and analyze its molecular mechanisms, looking for the potential molecular targets for the treatment or alleviation of obesity-related metabolic disorders.

## Figures and Tables

**Table 1:** The composition of the diet used in this study.

Diet	Company	Catalog	Cal Density (kcal/g)	Fat (%)		Protein (%)	Carbohydrate (%)
NFD	Research Diets	AIN-93G	3.72	11		34	55
				soybean oil/2.8%			
HFD	Research Diets	D12492	5.24	60		20	20
				soybean oil/3.23%	Lard/31.66%		

**Table 2:** List of primer sequences used in this study.

Gene Name	Forward (5′- > 3′)	Reverse (5′- > 3′)
*ACC*	GATGAACCATCTCCGTTGGC	CCCAATTATGAATCGGGAGTGC
*Adrb3*	GGCCCTCTCTAGTTCCCAG	TAGCCATCAAACCTGTTGAGC
*Atgl*	CTGAGAATCACCATTCCCACATC	CACAGCATGTAAGGGGGAGA
*C/EBPa*	CAAGAACAGCAACGAGTACCG	GTCACTGGTCAACTCCAGCAC
*Cidea*	TGCTCTTCTGTATCGCCCAGT	GCCGTGTTAAGGAATCTGCTG
*CPT1a*	CACTGCAGCTCGCACATTAC	CCAGCACAAAGTTGCAGGAC
*Dgat2*	GCGCTACTTCCGAGACTACTT	GGGCCTTATGCCAGGAAACT
*GAPDH*	TGTGTCCGTCGTGGATCTGA	CCTGCTTCACCACCTTCTTGA
*Hsl*	TCCTCAGAGACCTCCGACTG	ACACACTCCTGCGCATAGAC
*Plin1*	CAAGCACCTCTGACAAGGTTC	GTTGGCGGCATATTCTGCTG
*PPARg*	GGAAGACCACTCGCATTCCTT	TCGCACTTTGGTATTCTTGGAG
*UCP1*	GCTTTGCCTCACTCAGGATTGG	CCAATGAACACTGCCACACCTC

## Data Availability

Data are contained within the article.
